# Lead Pipes Poisonous

**Published:** 1873-02

**Authors:** 


### LEAD PIPES POISONOUS.

No doubt many persons have been injured by drinking water, or using it in cooking, which has passed through lead pipes; but the injury has been greatly exaggerated. Otherwise, every human being in New York and other large cities would have been dead long ago. Yet cases of fatal poisoning will continue to occur from defective laying of the pipes, from using the water too soon after they are laid, from not letting the water run off a minute after none has been drawn for ten hours or more, and also from the chemical constituents of some waters, which have a decomposing power over lead. For these reasons, the only perfectly safe water conduits are stone, wood, and glass, or lead lined with glass. This is lately claimed to be done in this city, and if a reality, and practicable at a moderate expense, the inventors deserve to realize a large fortune for their intelligence, energy, and enterprise in accomplishing so great a public good.

